# Synthesis and Characterization of Multiple-Cation Rb(MAFA)PbI_3_ Perovskite Single Crystals

**DOI:** 10.1038/s41598-019-38947-3

**Published:** 2019-02-14

**Authors:** Hyojung Kim, Hye Ryung Byun, Mun Seok Jeong

**Affiliations:** 1 Department of Energy Science, Sungkyunkwan University, Suwon, 16419 Republic of Korea; 2 Center for Integrated Nanostructure Physics, Institute for Basic Science (IBS), Suwon, 16419 Republic of Korea

**Keywords:** Materials science, Optics and photonics

## Abstract

We synthesized multiple-cation Rb(MAFA)PbI_3_ perovskite single crystals for the first time. The effect of Rb^+^ substitution was systemically investigated, and the addition of 1.5 M 5% RbI was the optimum condition to obtain high-quality Rb(MAFA)PbI_3_ single crystals. Lattice shrinkage occurred in the Rb(MAFA)PbI_3_ single crystal because of the small ionic radius of Rb^+^, resulting in blue-shifted absorption and photoluminescence (PL) peaks. The 1.5 M 5% RbI-added (MAFA)PbI_3_ single crystal showed the longest carrier lifetime of 18.35 ns, exhibiting the highest photoresponse than other crystals. We believe that this work will provide a basic insight into the mixed-cation perovskite single crystals for the future optoelectronic applications.

## Introduction

Hybrid organic–inorganic perovskites with an ABX_3_ structure (where A is a monovalent cation such as methylammonium (CH_3_NH_3_^+^; MA) and formamidinium (CH_3_(NH_2_)^2+^; FA); B and X refer to divalent cations (Pb^2+^, Sn^2+^) and halogen anions (I^−^, Br^−^, Cl^−^), respectively) have been considered as promising optoelectronic materials because of their excellent optical and electrical properties^1–5^. However, these perovskites suffer from low stability in air because of the inherent instability of their organic cations^6,7^, which limits their practical applications. To overcome this limitation, compositional engineering of these perovskites has been intensively studied^6,8–10^. For example, Zhang *et al*. obtained better crystal quality and enhanced device performance by adding 10% FA^+^ to MAPbI_3_ solar cells^11^. Wang *et al*. also obtained similar results when they mixed MA^+^ and FA^+^ cations in a ratio of 7:3^9^. Recently, Saliba *et al*. added Cs cations to MAFA perovskites and achieved great solar cell performance with improved thermal stability^12^. Zhang *et al*. also investigated the effect of incorporating Cs^+^ in perovskites and demonstrated that triple-cation solar cells exhibit better performance than do single-cation solar cells in terms of thermal and humidity stabilities^13^. More recently, Rb has been introduced as the fourth candidate for multiple-cation perovskites^14,15^. Interestingly, based on the Goldschmidt tolerance factor, perovskite structures cannot be formed with Rb^+^ because of its small ionic radius (152 pm)^14^. However, with the incorporation of small amounts Rb^+^, high photovoltaic efficiency and low hysteresis can be achieved because Rb^+^ can significantly suppress the yellow phase in perovskite films^14,16–19^. Despite these rapid improvements in device performance, the use of polycrystalline perovskites is still limited because of their grain boundary issues. Shao *et al*. reported that grain boundaries acts as ion migration channels, resulting in current hysteresis in perovskite optoelectronic devices^20^. Moreover, Wang *et al*. studied the morphology-dependent degradation of polycrystalline perovskite films and found that the grain boundaries in perovskites accelerate their degradation process because of the diffusion of moisture through them^21^. This limitation can be overcome using perovskite single crystals, which offer the following advantages: the absence of grain boundaries, low trap density, and excellent air stability^2,22,23^. However, the effect of mixed cations on single crystals remains nearly unexplored. To the best of our knowledge, there has been no report on triple-cation perovskite single crystals, especially those with Rb^+^ incorporation.

In this study, we synthesized and characterized multiple-cation Rb(MAFA)PbI_3_ perovskite single crystals for the first time. X-ray diffraction (XRD) and time-of-flight secondary ion mass spectroscopy (ToF-SIMS) analyses confirmed the incorporation of Rb^+^ ions into the perovskite single crystal. We found that an Rb concentration of 1.5 M (5%) was optimum to obtain Rb(MAFA)PbI_3_ single crystals with high Rb ratios. The effect of the addition of Rb^+^ on the perovskite single crystal was systemically studied via UV-Vis absorption and photoluminescence (PL) spectroscopy. Finally, based on the time-resolved PL (TRPL) and photocurrent data, we proved that the addition of Rb^+^ increased the carrier lifetime and photoresponse of the perovskite single crystal. We believe that this work will provide a basic insight into the structure of mixed-cation perovskite single crystals for future applications.

## Results and Discussion

**Figure 1:**
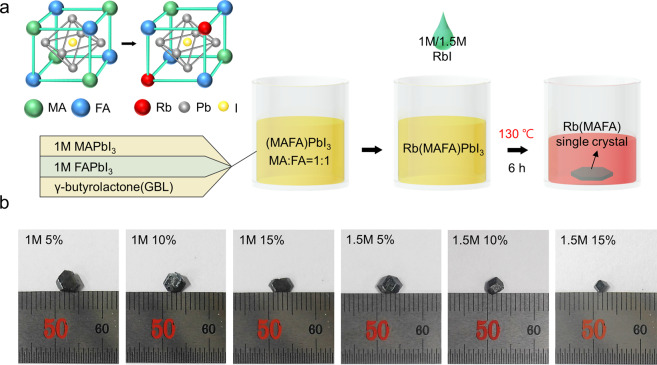
Synthesis of the Rb(MAFA)PbI_3_ single crystals with various RbI concentrations. (**a**) Schematics of the Rb(MAFA)PbI_3_ structure and the detailed ITC process. (**b**) Photographs of the Rb(MAFA)PbI_3_ single crystals with various RbI contents.

The Rb(MAFA)PbI_3_ perovskite single crystals were grown using an inverse temperature crystallization (ITC) method^24^. The structure of Rb(MAFA)PbI_3_ and the detailed growth process are illustrated in Fig. 1a. (MAFA)PbI_3_ has a cubic structure at room temperature^23,25^, and smaller Rb^+^ ions randomly replace the organic cations^26^, MA^+^, and FA^+^, reducing its cubic volume. Generally, the solubility of common precursor salts increases with an increase in temperature. Perovskite salts, on the other hand, show reduced solubilities in solvents such as γ-butyrolactone (GBL) at elevated temperatures^24^. Hence, we synthesized Rb(MAFA)PbI_3_ single crystals with different Rb ratios using a GBL-based ITC method. The filtrates of 1 M MAPbI_3_ and FAPbI_3_ solutions were mixed in a 1:1 ratio. To this mixture, 1 and 1.5 M RbI solutions (5, 10, and 15%) were added (see Supplementary Information for details). After storing the mixed solution in a convection oven at 130 °C for 6 h, the dodecahedral Rb(MAFA)PbI_3_ single crystals were grown, as shown in Fig. 1b. The shape of single crystal highly depends on the growth solution; both dodecahedral- and cubic-shaped single crystal of the same composition can be grown by varying the growth solution condition^27^. The size of the crystals depended on the RbI content. The average crystal size was found to be 3–5 mm. We further proved the absence of grain boundaries in the Rb(MAFA)PbI_3_ single crystals via scanning electron microscopy (SEM) (Figure S1). As mentioned earlier, the absence of grain boundaries is one of the most important advantages of single crystalline perovskites over polycrystalline perovskites.

**Figure 2:**
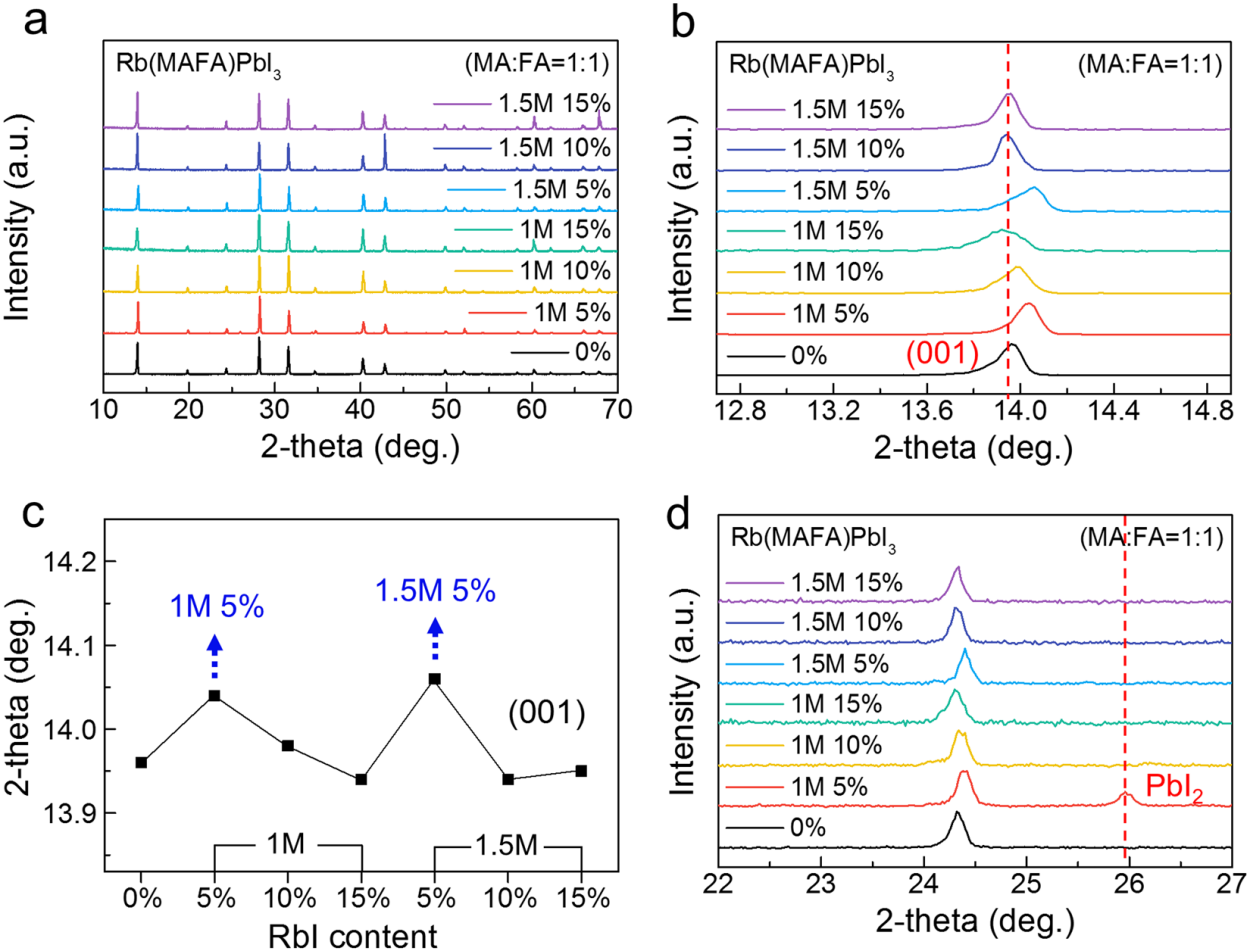
Powder XRD analysis of the Rb(MAFA)PbI_3_ single crystals. (**a**) XRD patterns of the Rb(MAFA)PbI_3_ perovskite single crystals with different Rb ratios. (**b**) XRD peak shifts in the region of the (001) diffraction and (**c**) shifted peak values with the increasing RbI concentration. (**d**) XRD spectra of Rb(MAFA)PbI_3_ near 25.9° attributed to the (020) diffraction of PbI_2_.

In order to investigate the effect of the Rb ratio on the structure of the Rb(MAFA)PbI_3_ single crystals, we carried out a powder XRD analysis on them. The diffraction peak at 13.9° in Fig. 2a corresponds to the (001) plane of the cubic perovskite structure^25^, which means that the (MAFA)PbI_3_ single crystal had a cubic phase before the addition of Rb^+^. We analyzed the changes in the XRD patterns of the single crystal as a function of the RbI concentration. However, no additional peak was observed after the addition of Rb^+^. This indicates that the Rb(MAFA)PbI_3_ single crystal retained the original cubic phase of (MAFA)PbI_3_ even after the addition of Rb^+^. For more details, we zoomed the (001) plane in Fig. 2b, and it was found that the diffraction peak shifted to higher angles depending on the Rb ratio. We fitted the diffraction peak at 13.9° to Lorentzian function, and the average FWHM value of Rb(MAFA)PbI_3_ perovskite crystals was about 0.11°, which is sharper than that of the perovskite film^26^. Generally, FWHM of XRD profiles gives information about crystal quality of materials^28^; thus, we assumed that the single crystalline perovskites showed sharper FWHM than that of polycrystalline perovskites as a result of no grain boundaries. Further, we compared the FWHM values with the Rb^+^ addition, and the 1.5 M 5% RbI-added single crystal showed slightly broader FWHM of 0.13°. Considering the small size of Rb^+^, the broader FWHM can also be understood by the effect of crystal distortion in Rb(MAFA)PbI_3_ single crystals. We plotted the shifted peak values as a function of the RbI content (Fig. 2c) and observed a significant peak shift when 5% RbI was added (blue arrow). Recently, Shi *et al*. have reported similar XRD peak shifts for Rb^+^-incorporated FAPbBr_3_ films. They reported that the diffraction peak shifted toward wider angles because of the Rb^+^-induced crystal distortion due to the small ionic radius (152 pm) of Rb^+^ (compared to that of FA^+^ (253 pm))^29^. In other words, the diffraction peak shift increased with an increase in the Rb^+^ content. Thus, by monitoring the shifted peak values in Fig. 2c, we assumed that the addition of 5% RbI was optimum. Further, we compared the other diffraction peaks and observed the remaining PbI_2_ peak at 25.9° from the 1 M 5% RbI-added crystal, as shown in Fig. 2d. Although the addition of 5% RbI was optimum the addition of 1.5 M RbI resulted in better crystal quality than that obtained when 1 M RbI was added. Hence, it can be stated that the addition of 1.5 M 5% RbI was optimum to synthesize high-quality Rb(MAFA)PbI_3_ single crystals with a high Rb^+^ concentration.

**Figure 3:**
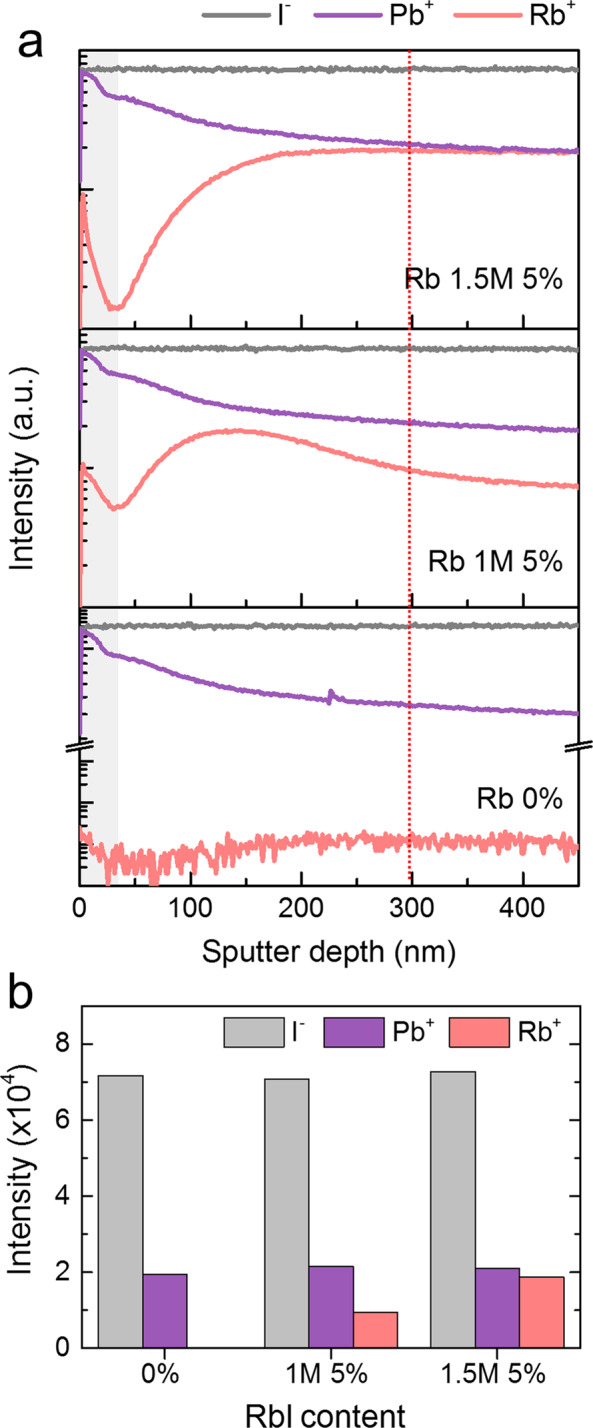
ToF-SIMS analysis of the Rb(MAFA)PbI_3_ single crystals. (**a**) ToF-SIMS depth profiles of I^−^ (126.9 m/z), Pb^+^ (207.9 m/z), and Rb^+^ (84.9 m/z) ions from the Rb(MAFA)PbI_3_ single crystals as a function of the RbI content. (**b**) The intensity of emitted I^−^, Pb^+^, and Rb^+^ ions as a function of the RbI content.

To demonstrate the Rb^+^ substitution, we carried out the ToF-SIMS analysis of the single crystals with various RbI concentrations. We selected I^−^ (126.9) and Pb^+^ (207.9) for comparison and monitored the emitted Rb^+^ (84.9) signals from the Rb(MAFA)PbI_3_ single crystals as a function of the RbI content. Figure 3a shows the ToF-SIMS depth profiles of the secondary ions emitted from the Rb(MAFA)PbI_3_ single crystals. As shown in Fig. 3a, the intensity of the Rb^+^ signal changed significantly with an increase in the Rb ratio. We also plotted the changes in the intensity of the emitted I^−^, Pb^+^, and Rb^+^ ions as a function of the RbI content at a sputtering depth of 300 nm (Fig. 3b). We found that the intensity of both I^−^ and Pb^+^ did not change; however, that of Rb^+^ increased gradually with an increase in the RbI content. In particular, the 1.5 M 5% RbI-added single crystal showed the largest number of Rb cations. However, at higher RbI contents of 10% or 15%, the Rb cation substitution ratio decreased significantly (Fig. S2). This indicates that the addition of 1.5 M 5% RbI is the optimum condition to obtain Rb(MAFA)PbI_3_ single crystals with high Rb^+^ content. This is consistent with the XRD results shown in Fig. 2.

**Figure 4:**
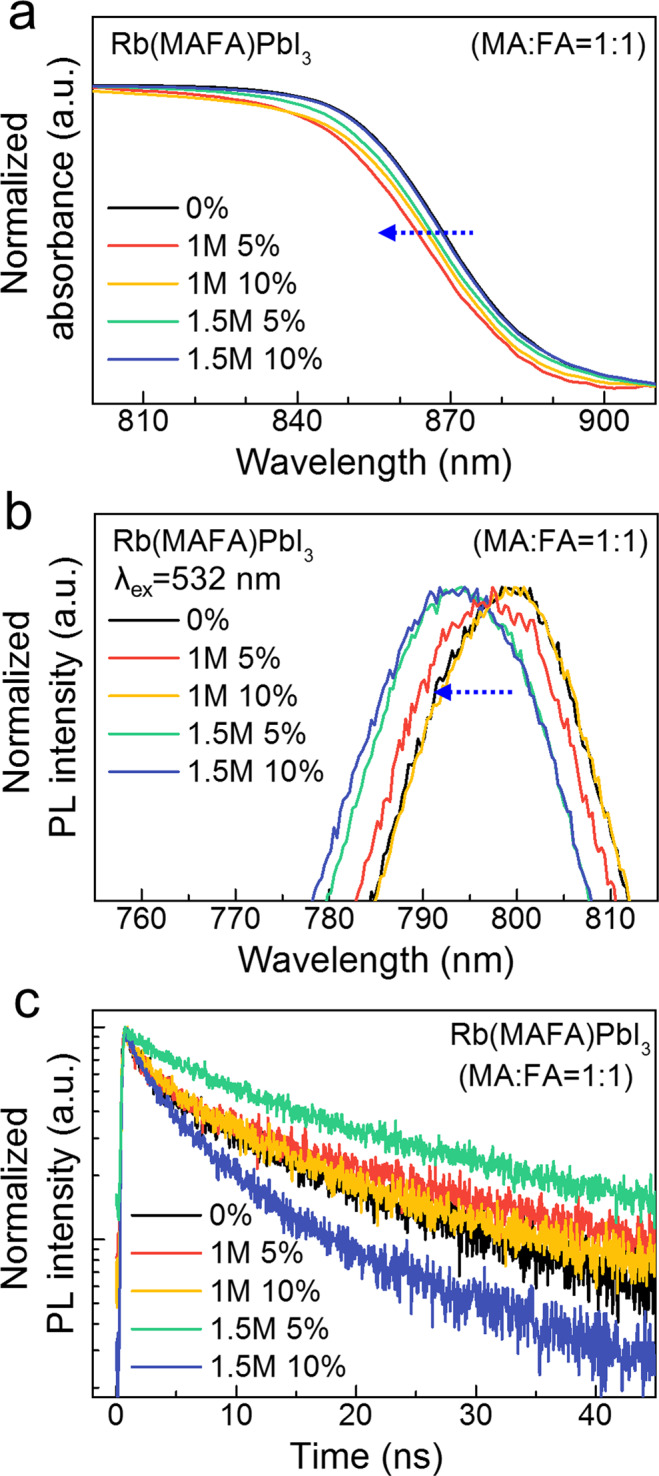
Optical properties of the Rb(MAFA)PbI_3_ single crystals. (**a**) Linear absorption and (**b**) PL spectra of the Rb(MAFA)PbI_3_ single crystals with various RbI contents. (**c**) TRPL decay profile of the Rb(MAFA)PbI_3_ single crystals with different Rb ratios.

We further investigated the effect of the RbI content on the optical properties of the Rb(MAFA)PbI_3_ single crystals. Linear absorption and PL spectra of the single crystals with various Rb contents are shown in Fig. 4a,b. Here, we have not shown the data for 15% RbI addition because of the small size and low yield of the corresponding single crystal (Fig. S3). Basically, in the absence of Rb, the (MAFA)PbI_3_ perovskite single crystal showed an optical band edge and a PL peak at around 870 and 800 nm, respectively. The above-band gap PL characteristics of perovskite single crystals have been reported^2,30^; however, the origin of this effect is still under debate. After the addition of Rb^+^ into the (MAFA)PbI_3_ single crystal, the absorption and PL peaks slightly blue-shifted by about 5.17 and 6.29 nm, respectively. According to previous reports, metal-halide-metal bonds, which are related to the electronic band structure of perovskites, are indirectly affected by the lattice shrinkage due to Rb^+^ substitution, leading to a down-shifted valence band maximum^29^. As a result, the Rb(MAFA)PbI_3_ perovskite single crystals showed blue-shifted absorption and PL peaks. The expanded absorption spectrum to the near-infrared region is needed to be addressed because the solar cell efficiency would be improved owing to a significant amount of solar energy in this wavelength range. The average thickness of Rb(MAFA)PbI_3_ crystals was 1.48 mm, and the optical band edges were located at approximately 865 nm for all crystals.

The TRPL measurements of the single crystals with various Rb ratios were also carried out. Figure 4c shows the normalized PL decay curves of the Rb(MAFA)PbI_3_ single crystals with various Rb ratios. We extracted two decay components fitted to a bi-exponential function and calculated the average lifetime of the crystals (see Supplementary Information for details). The (MAFA)PbI_3_ perovskite single crystal showed an average carrier lifetime of 13.01 ns (Table 1). In contrast, the 5% RbI-added single crystal showed a longer carrier lifetime than the other crystals. The 1.5 M 5% RbI-added (MAFA)PbI_3_ single crystal showed the longest carrier lifetime of 18.35 ns. We expect that Rb^+^-based perovskite single crystals have low trap-state densities and long carrier diffusion lengths through the remarkably increased lifetime in the TRPL results. From these results, it can be stated that the addition of Rb^+^ increases the carrier lifetime of perovskite single crystals, which is beneficial for future applications.

**Table 1:** The calculated average carrier lifetimes of the Rb(MAFA)PbI_3_ single crystals as a function of the RbI content.

RbI content	t_average_ (ns)
0%	13.01
1 M 5%	14.39
1 M 10%	11.72
1.5 M 5%	18.35
1.5 M 10%	7.57

Lastly, we have carried out additional experiments to disclose the practical effect with Rb^+^ addition as shown in Fig. S4. We studied photocurrent properties of the Rb(MAFA)PbI_3_ perovskite single crystals. For the photocurrent measurement, the 100-nm-thick Au electrodes were thermally evaporated on the top of the Rb(MAFA)PbI_3_ crystals with an average channel length of 622 μm. The device was characterized using a 689 nm excitation source, and we found that the 1.5 M 5% RbI-added (MAFA)PbI_3_ single crystal exhibited the highest photoresponsivity than other crystals. This result indicates that the addition of Rb^+^ also has a positive effect on the electrical properties of perovskite single crystals.

## Conclusions

We successfully synthesized multiple-cation Rb(MAFA)PbI_3_ perovskite single crystals for the first time. The effect of Rb^+^ substitution on the properties of the perovskite single crystals was systemically investigated using XRD and ToF-SIMS measurements. It was found that the addition of 1.5 M 5% RbI was the optimum condition for the formation of high-quality Rb(MAFA)PbI_3_ single crystals. Lattice shrinkage occurred in the Rb(MAFA)PbI_3_ single crystal because of the small ionic radius of Rb^+^. As a result, blue-shifted absorption and PL peaks were observed upon Rb^+^ substitution. The 1.5 M 5% RbI-added (MAFA)PbI_3_ perovskite single crystal showed the longest carrier lifetime of 18.35 ns, with the highest photoresponsivity compared to other crystals. We believe that the fundamental understanding and optimization of the Rb^+^-incorporated perovskite single crystals will introduce a new platform for the development of multiple cation single crystals for future optoelectronic applications.

## Supplementary information

Synthesis and Characterization of Multiple-Cation Rb(MAFA)PbI<sub>3</sub> Perovskite Single Crystals
