# Author Correction: Synthesis and Characterization of Multiple-Cation Rb(MAFA)PbI_3_ Perovskite Single Crystals

**DOI:** 10.1038/s41598-023-33296-8

**Published:** 2023-04-13

**Authors:** Hyojung Kim, Hye Ryung Byun, Mun Seok Jeong

**Affiliations:** 1 Department of Energy Science, Sungkyunkwan University, Suwon, 16419 Republic of Korea; 2 Center for Integrated Nanostructure Physics, Institute for Basic Science (IBS), Suwon, 16419 Republic of Korea

Correction to: *Scientific Reports* 10.1038/s41598-019-38947-3, published online 14 February 2019

This Article contains an error in Figure 1, panel (a), where the yellow atom is incorrectly labelled as “I”, and the grey atom is incorrectly labelled as “Pb”.

The correct Figure 1 and accompanying legend appear below.

**Figure 1:**
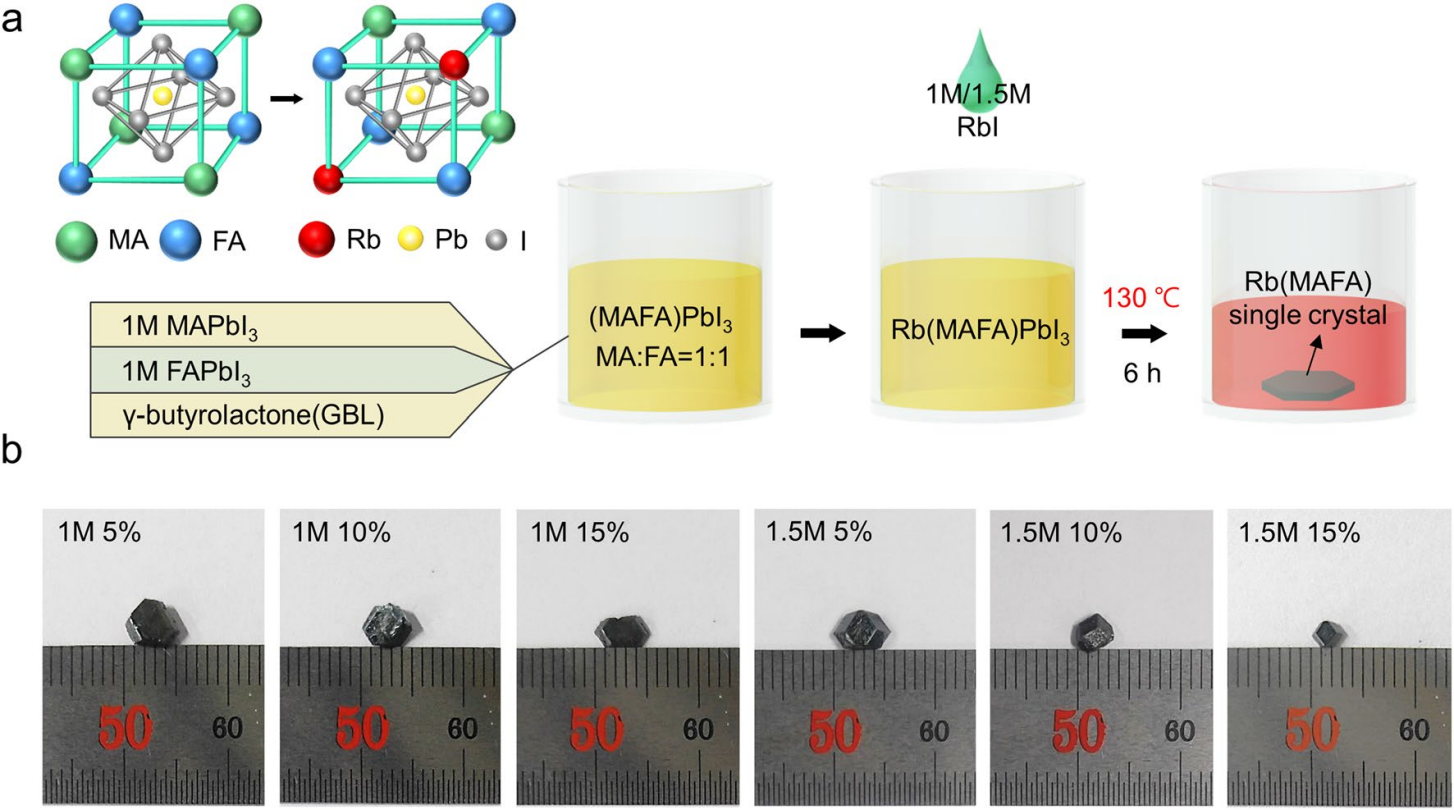
Synthesis of the Rb(MAFA)PbI_3_ single crystals with various RbI concentrations. (**a**) Schematics of the Rb(MAFA)PbI_3_ structure and the detailed ITC process. (**b**) Photographs of the Rb(MAFA)PbI_3_ single crystals with various RbI contents.

