# Supplementary material for: Synthesis and Characterization of Multiple-Cation Rb(MAFA)PbI3 Perovskite Single Crystals
Source: Sci Rep. 2019 Feb 14;9:2022. doi: 10.1038/s41598-019-38947-3 (PMC6376001; doi:10.1038/s41598-019-38947-3)
Supplement: Supplementary file 1 — Synthesis and Characterization of Multiple-Cation Rb(MAFA)PbI3 Perovskite Single Crystals [file 41598_2019_38947_MOESM1_ESM.docx]

**Supplementary Information**

**Synthesis and Characterization of Multiple**-**Cation Rb(MAFA)PbI_3_ Perovskite Single Crystals**

Hyojung Kim,^1,2^ Hye Ryung Byun,^1,2^ and Mun Seok Jeong^1,2^

^1^ Department of Energy Science, Sungkyunkwan University, Suwon, 16419, Republic of Korea.

^2^ Center for Integrated Nanostructure Physics, Institute for Basic Science (IBS), Suwon, 16419, Republic of Korea.

**Experimental Section**

***Chemicals*:** Methylammonium iodide (MAI) and formamidinium iodide (FAI) were purchased from GreatCell Solar (Australia), and rubidium iodide (RbI) and γ-butyrolactone (GBL, ≥99%) were purchased from Sigma Aldrich. Lead iodide (PbI_2_, ≥99.99%) was purchased from Alfa Aesar.

***Synthesis of the Rb(MAFA)PbI_3_ single crystals:*** Rb**(**MAFA)PbI_3_ perovskite single crystals were grown using an inverse temperature crystallization (ITC) method with some modifications. The MAPbI_3_ (containing 1 M MAI and PbI_2_ in GBL) and FAPbI_3_ (containing 1 M FAI and PbI_2_ in GBL) solutions were mixed in a 1:1 ratio. To this mixture, 1 and 1.5 M RbI solutions (containing RbI in GBL) were added (5% RbI:(MAFA)PbI_3_ = 5:95 (v/v), i.e., Rb_5_(MAFA)_95_PbI_3_; 10% RbI:(MAFA)PbI_3_ = 10:90 (v/v), i.e., Rb_10_(MAFA)_90_PbI_3_; 15% RbI:(MAFA)PbI_3_ =15:85 (v/v), i.e., Rb_15_(MAFA)_85_PbI_3_). All solutions were heated at 70°C for 24 h (in ambient condition with 35–40% relative humidity) and filtered using a hydrophobic polytetrafluoethylene (PTFE-D) filter with 0.2 µm pore size. The filtrate of mixed Rb(MAFA)PbI_3_ solution (3 mL) was stored in a convection oven at 130 °C for 6 h.

***Characterization:*** Field-emission scanning electron microscopy (FESEM, JSM7000F, JEOL) was performed to investigate the surface morphologies of the Rb**(**MAFA)PbI_3_ single crystals. Powder X-ray diffraction (XRD) analysis was carried out using a X-ray diffractometer (Rigaku, SmartLab) with Cu-K_α_ radiation (λ = 1.54059 Å) in the Bragg-Brentano focusing geometry at 45 kV and 200 mA with a tube. The time-of-flight secondary ion mass spectroscopy (ToF-SIMS) depth profiles were collected using a TOF analyzer (TOF-SIMS 5, Ion-TOF GmbH), equipped with a 25 keV Bi^+^ beam for the analysis and a 1 keV Cs^+^ and O^2^ source for the sputtering. Linear absorption spectra of the Rb**(**MAFA)PbI_3_ single crystals were measured by using a commercial spectrometer (V-670, JASCO). The photoluminescence (PL) and time-resolved PL (TRPL) measurements were conducted using a confocal microscope system (NTEGRA SPECTRA, NT-MDT) with a 100× objective lens (NA 0.7). For the PL measurement, a 532 nm solid-state laser was used (1.94 μW), and spectra were collected by using a thermoelectrically cooled CCD detector with an exposure time of 0.1 s. For the TRPL measurement, a 405 nm pulsed laser with a repetition rate of 20 MHz was used, and a high-speed photomultiplier tube detector (PMC-100, Photonic Solutions) was applied for the time-correlated single photon counting system.

***Average carrier lifetime calculation:*** The TRPL decay profile was fitted to a bi-exponential function given by

$y=y_{0}+a_{1}*exp(-x/t_{1})+a_{2}*exp(-x/t_{2})$, (1)

where y_0_ is an offset, *a* is a weight constant, and *t*_1_, *t*_2_ are time decay constants.

We calculated the average amplitudes *A*_1_, *A*_2_ and the average lifetime as follows:

$$A_{1}\left( \% \right)=\frac{a_{1}}{a_{1}+a_{2}}\times100$$

(2)

$$A_{2}\left( \% \right)=\frac{a_{2}}{a_{1}+a_{2}}\times100$$

$$Average lifetime (ns)=\frac{A_{1}t_{1}^{2}+A_{2}t_{2}^{2}}{A_{1}t_{1}+A_{2}t_{2}}$$

**
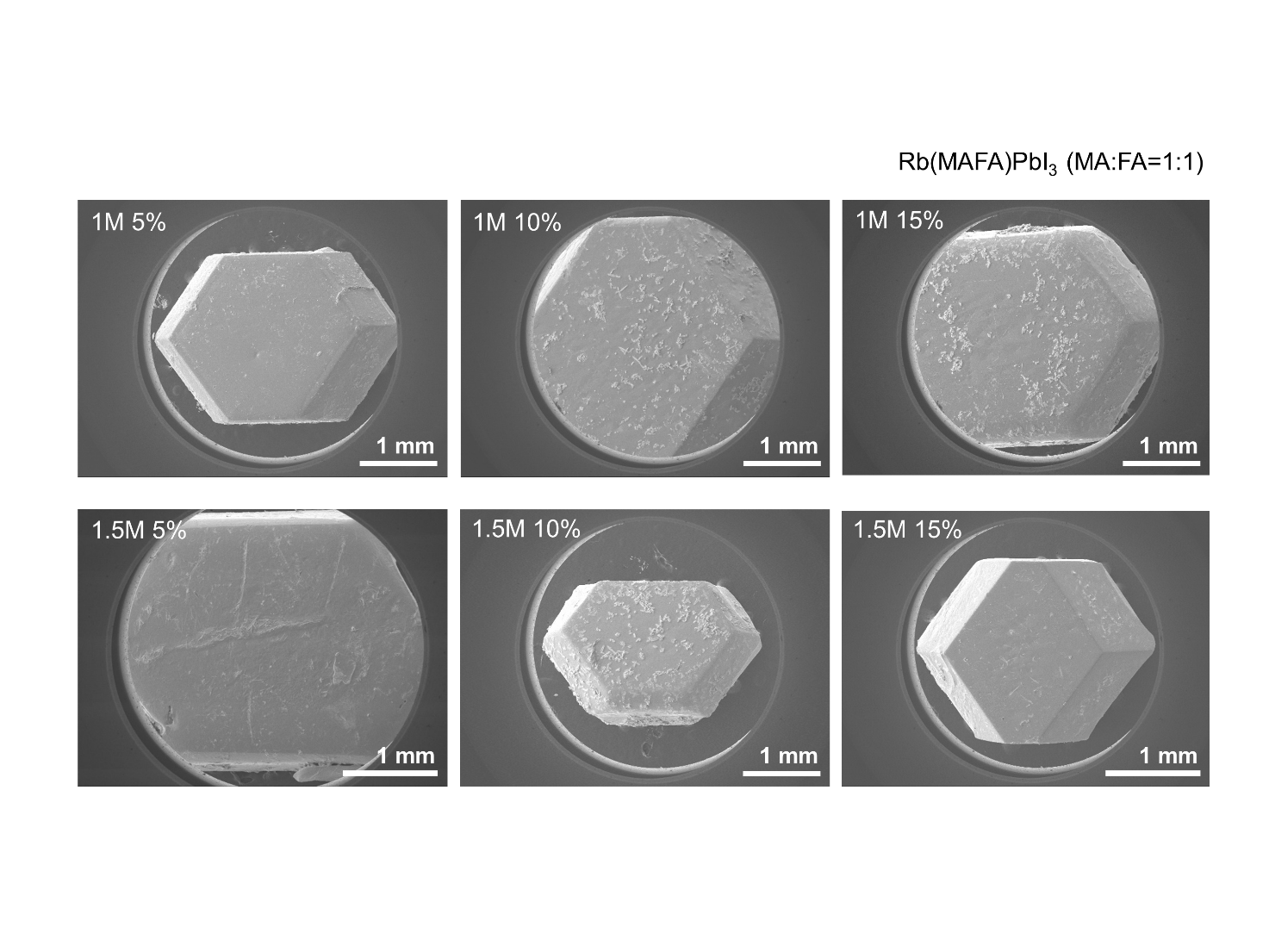
**

**Figure S1.** Top-view SEM images of the Rb(MAFA)PbI_3_ single crystals with different Rb ratios.

**
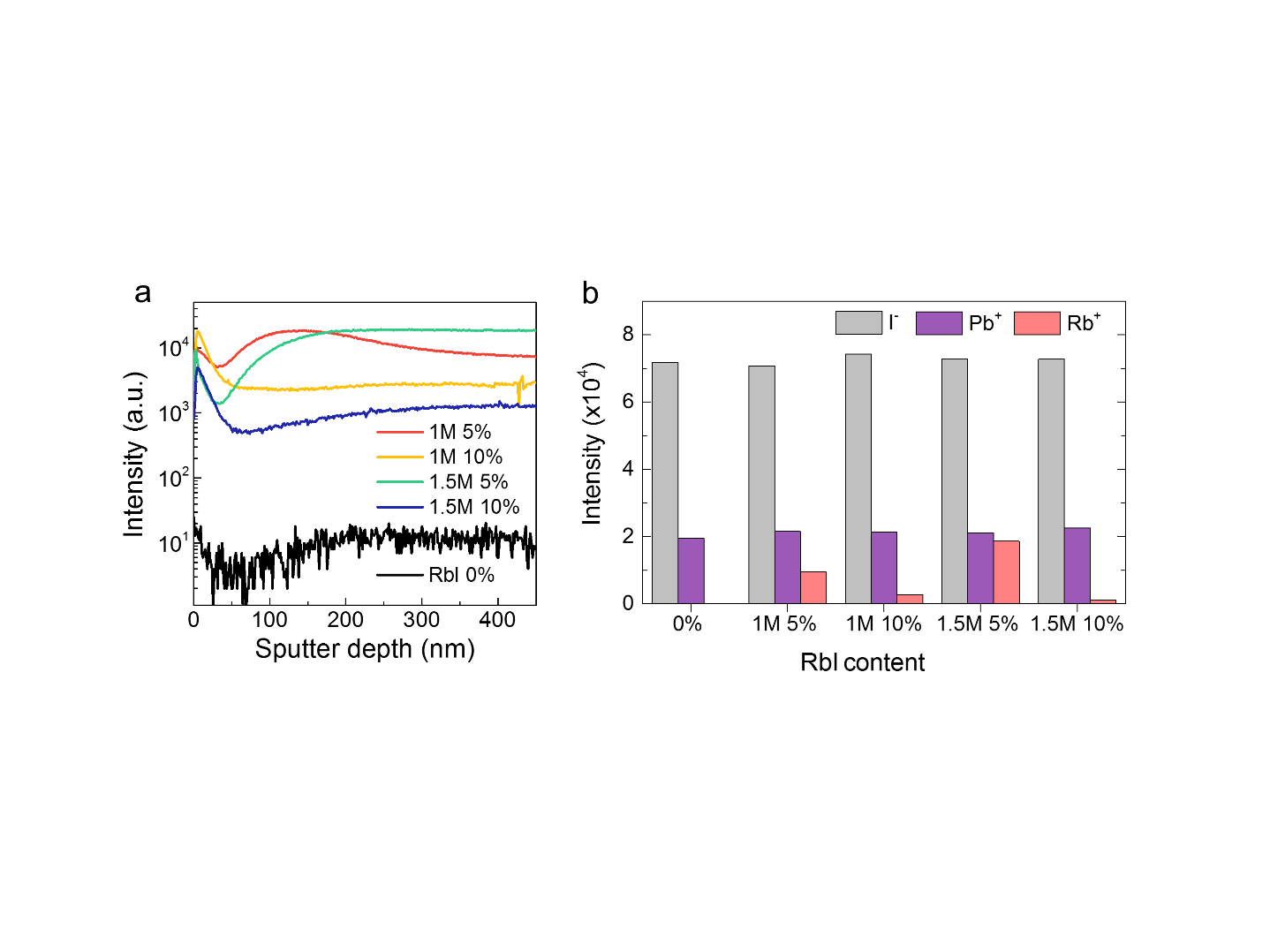
**

**Figure S2.** (a) ToF-SIMS depth profiles of Rb^+^ (84.9 m/z) ion from the Rb(MAFA)PbI_3_ single crystals as a function of the RbI content. (b) The intensity of emitted I^-^, Pb^+^, and Rb^+^ ions as a function of the RbI content.

**
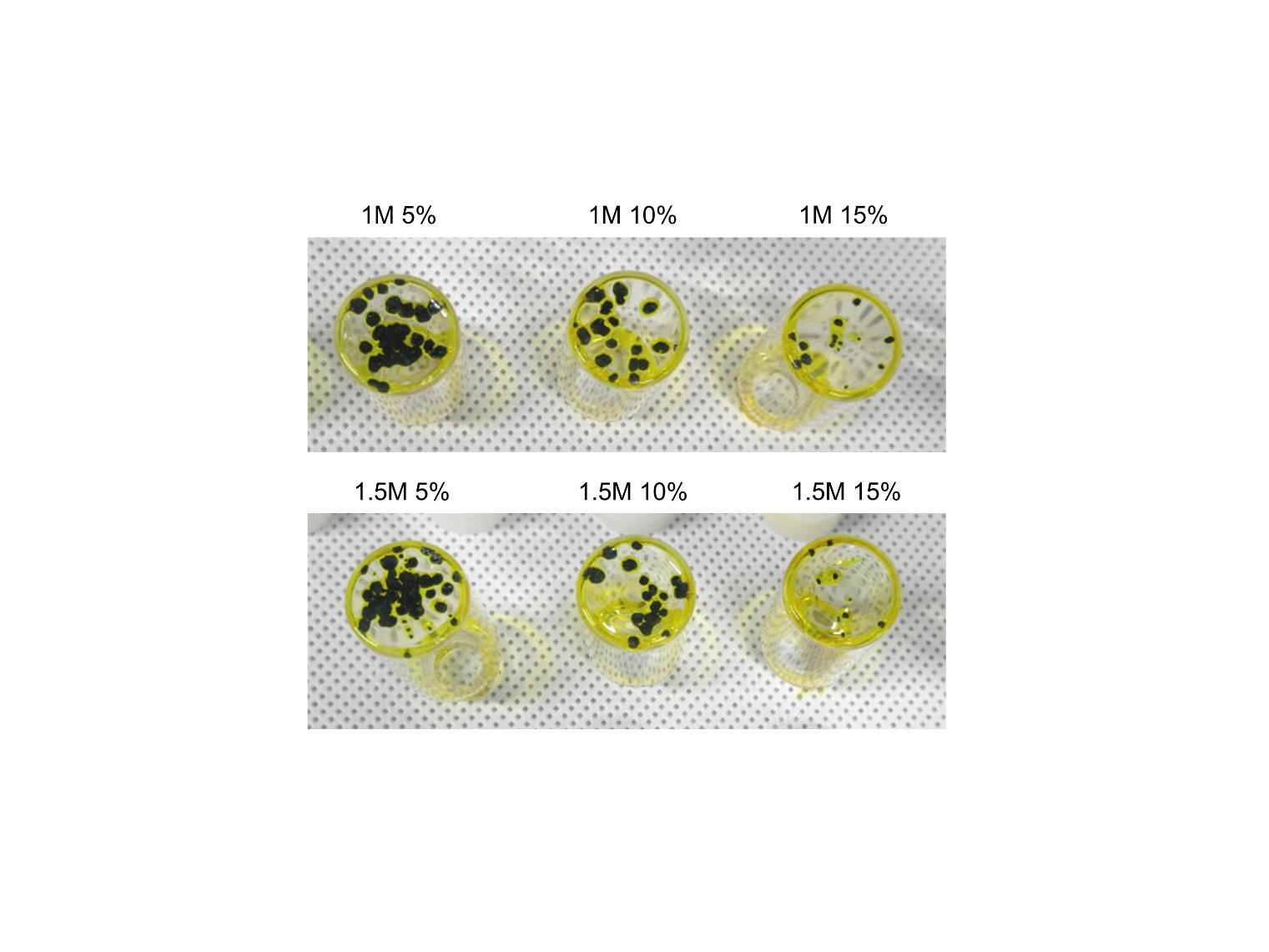
**

**Figure S3.** The yield of the Rb(MAFA)PbI_3_ single crystals with various RbI contents.


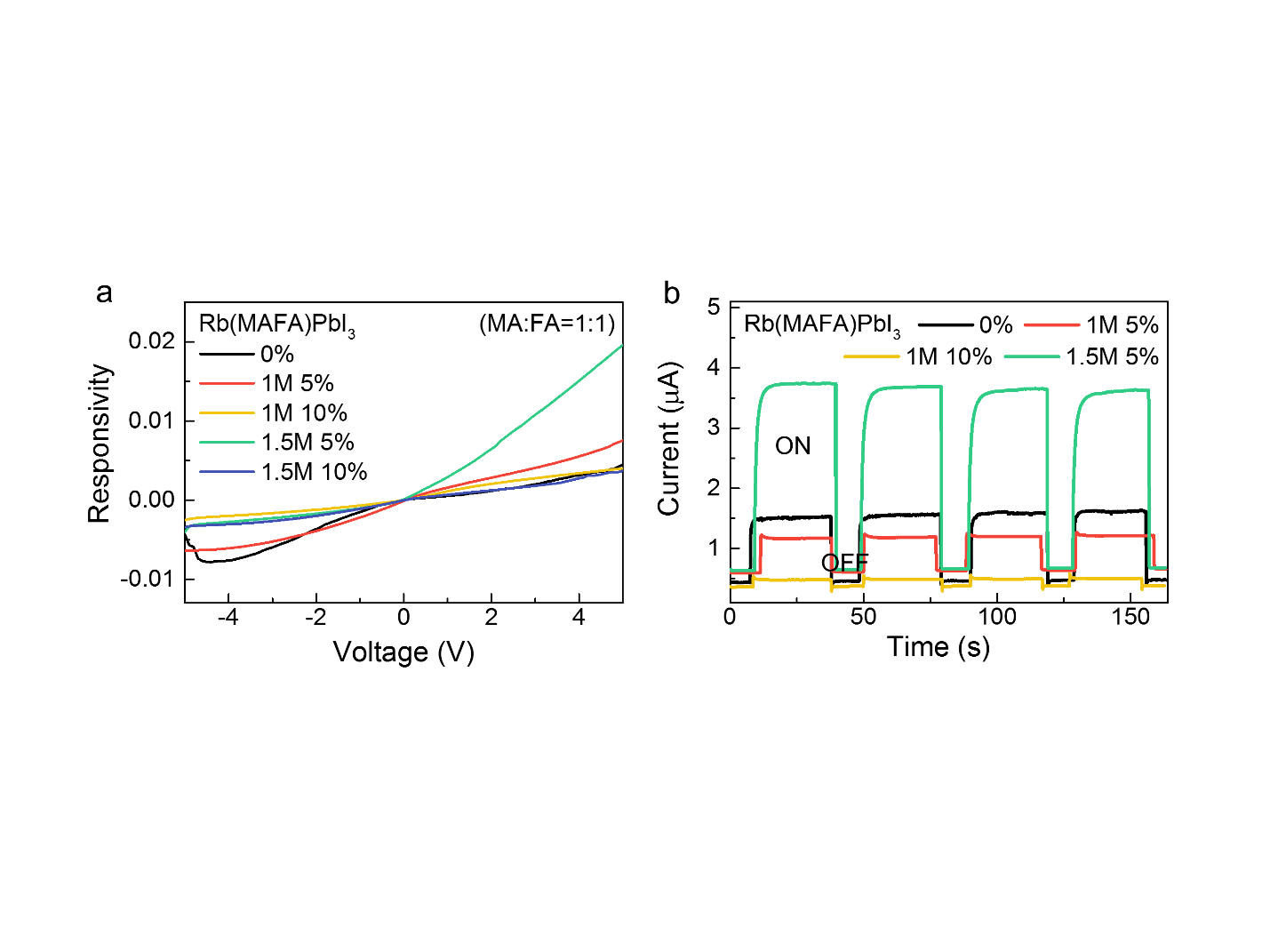


**Figure S4.** (a) Responsivities and (b) photocurrent responses of the Rb(MAFA)PbI_3_ single crystals with various Rb^+^ ratios.
